# Segmentation and Morphometric Analysis of Cells from Fluorescence Microscopy Images of Cytoskeletons

**DOI:** 10.1155/2013/381356

**Published:** 2013-05-12

**Authors:** Yoshihiro Ujihara, Masanori Nakamura, Hiroshi Miyazaki, Shigeo Wada

**Affiliations:** ^1^Department of Physiology, Kawasaki Medical School, 577 Matsushima, Kurashiki, Okayama 701-0192, Japan; ^2^Graduate School of Science and Engineering, Saitama University, Saitama, Saitama 338-8570, Japan; ^3^Graduate School of Engineering Science, Osaka University, Toyonaka, Osaka 560-8531, Japan

## Abstract

We developed a method to reconstruct cell geometry from confocal fluorescence microscopy images of the cytoskeleton. In the method, region growing was implemented twice. First, it was applied to the extracellular regions to differentiate them from intracellular noncytoskeletal regions, which both appear black in fluorescence microscopy imagery, and then to cell regions for cell identification. Analysis of morphological parameters revealed significant changes in cell shape associated with cytoskeleton disruption, which offered insight into the mechanical role of the cytoskeleton in maintaining cell shape. The proposed segmentation method is promising for investigations on cell morphological changes with respect to internal cytoskeletal structures.

## 1. Introduction

Advances in imaging modalities and technologies have enabled the investigation of organ structure-function relationships. Organ segmentation in medical image volumes allows three-dimensional (3D) anatomical visualization, leading to quantitative estimates of organ structures and the development of anatomically realistic computational models used in biology, physiology, and medicine. The same approach can be applied to analyze cell structure-function relationships.

Cell functions, including growth, differentiation, motility, and apoptosis, are associated with cell shape and structure [1–4]. Cells change shape by altering their internal structures, mainly the cytoskeleton, in response to changes in the mechanical environment. Mechanical stimuli acting on the extracellular matrix are transmitted to the cell via transmembrane adhesion receptors, including integrins, which induce cytoskeleton rearrangement, allowing the cell to recover mechanical balance internally and with respect to the extracellular matrix [5]. This dynamic rearrangement induces changes not only in the 3D shape but also in the function of cells [6]. Therefore, relating cell morphology and internal cytoskeletal structure is important to gain a better understanding of cell mechanics.

Cell morphology is characterized by cell length, diameter, height, projection area, and volume [7–12]. Although these are useful for classifying cell shape, the three-dimensionality of cells often leads to misinterpretations. For example, a circular cone cannot be distinguished from different 3D bodies with the same projection area and height, such as cylindrical columns or ellipsoidal bodies. Therefore, the 3D shape of a cell must be known before characterizing its morphology.

Microscopy imaging techniques for cell observation include phase contrast, differential interference contrast, scanning electron, atomic force, and confocal laser scanning microscopy. Confocal laser scanning microscopy is superior in illuminating intracellular structures, including the cytoskeleton, and providing a series of focused images of an object at selected depths. These serial images are analogous to those acquired by computational tomography and magnetic resonance imaging. Therefore, a similar methodology can be applied to segmenting cells using a series of images of a cell at selected depths.

Numerous segmentation techniques exist [13–19] for producing image series, which can be classified as either boundary or region based. Active contour models, such as snakes [14, 17], are the most popular frameworks for boundary-based methods. Active contour models have been developed for tracking deformable moving objects [14, 17]. This framework attempts to minimize an energy function associated with a contour and find its local minimum at the object boundary. The main drawbacks of active contour models are their high sensitivity to noise and strong dependency on the initial settings, making them insufficiently adaptive to topology, and the relatively high operator interactions. The level set methods [7] are an extension of active contour modeling. They are more robust, versatile, and efficient. They can define sharp corners, topological changes, and 3D effects. Although the computational cost is the same as that of other methods based on fast, narrowband adaptive techniques, the level set methods are slow to converge and can get stuck in local minima. In addition, they require considerable preparation to determine appropriate velocities for advancing the level set function. Region-growing methods [13, 20] are representative of the region-based approach. In the region-growing methods, the region of interest expands from a seed area by assimilating adjoining voxels with properties homogenous to those of the seed voxel according to specified parameters. In contrast to the active contour models, they have heavy computational costs and depend strongly on predefined homogeneity criteria. However, they are relatively insensitive to noise and can correctly separate regions based on defined criteria.

Segmenting cells using fluorescence microscopy images of the cytoskeleton is difficult using conventional methods. Active contour models are only efficient if they are close to the final solution. When studying a complex cell structure, these models often fail if the initial guesses are far from the solution. In addition, the cytoskeleton varies greatly between cells and creating a general model is difficult. Considering the color distinction between the cytoskeleton and other structures in fluorescent images, the region-growing method is the best option. However, in fluorescent images of cytoskeletons, intracellular noncytoskeletal regions, including the nuclear region, are the same color as extracellular regions. Consequently, differentiating them solely based on color and intensity is impossible. Therefore, it was necessary to develop a new method that allows us to quantify 3D shape of a cell and observe the structure and distribution of cytoskeletons in the same cell.

In this paper, we introduce a new method, based on the region-growing method, to segment cells using confocal fluorescence microscopy images of the cytoskeleton. Our motivation for the study is to quantitatively assess 3D shape of a cell while observing the structure and distribution of cytoskeletons in the same cell in order to understand how cytoskeletons contribute to maintaining cell morphology. To demonstrate the utility of the new method, we investigate the shape of intact cells and cells with disrupted actin filaments and microtubules that are the main components of cytoskeletons.

## 2. Materials and Methods

### 2.1. Cell Preparation

Fibroblasts obtained from rabbit patellar tendons and cultured in Dulbecco's modified Eagle's medium with 10% fetal bovine serum were used as described previously [12]. The cells (*P* = 8–12) seeded to coverslips were treated either with cytochalasin D (10 *μ*g/mL) for 3 h to disrupt actin filaments or colchicine (0.6 *μ*g/mL) for 2 h at 37°C to disrupt microtubules. Then, the cells were washed with phosphate-buffered saline (PBS) and fixed with 3.7% formaldehyde in PBS for 20 min at 4°C. The cells were washed with PBS again and permeabilized with PBS containing 0.1% Triton X-100 for 1 min. Next, the cells were washed in PBS and incubated with monoclonal anti-beta-tubulin antibody (Chemicon International, Temecula, CA, USA) in PBS containing 1% bovine serum albumin (BSA; Sigma-Aldrich, St. Louis, MO, USA) for 1 h at room temperature. After washing with PBS, the cells were incubated with Alexa Fluor 488-conjugated anti-mouse-IgG antibody (Molecular Probes, Eugene, OR, USA) in PBS containing 1% BSA for 45 min at room temperature for microtubule staining. To stain actin filaments, the cells were incubated with rhodamine-phalloidin (Molecular Probes) in PBS containing 1% BSA for 20 min at room temperature and then washed with PBS. The cells were washed again with PBS, and excess solution was removed from the coverslips. Next, one drop of the antifade reagent SlowFade Gold (Invitrogen, Carlsbad, CA, USA) was applied to each coverslip, which was mounted upside down on glass slides and fixed using melted paraffin wax.

The experiment followed the Guidelines for Animal Experiments, Graduate School of Engineering Science, Osaka University, Osaka, Japan.

### 2.2. Confocal Laser Scanning Microscopy

Samples were observed under a confocal laser scanning microscope (FV500; Olympus, Tokyo, Japan) with a 60× oil immersion objective (N.A. = 1.40; Olympus). An Ar laser set to 488 nm was used to excite samples labeled with Alexa Fluor 488 mouse IgG antibody, and a He-Ne laser set to 543 nm was used for the rhodamine-phalloidin-labeled samples. The laser power was controlled at 5.0% for the 488 nm and 50% for the 543 nm wavelengths. For the double-stained samples, images were acquired sequentially, allowing us to obtain images optimized for the labeling materials. The photomultiplier was adjusted to maximum sensitivity with no saturation. To reduce background noise and signals, image intensities less than the offset value were determined to be zero. Images were scanned at medium speed by default. If an image was too noisy, the scan speed was changed to slow. Images were formatted in a 24-bit colored bitmap with a resolution of 1024 × 768 pixels (0.24 × 0.24 *μ*m/pixel). The *z*-axis scan interval was 0.25 *μ*m.

Images acquired with the FV 500 photomultiplier are 12-bit grayscale. However, since the image format for 12-bit grayscale is unique to the FV 500, we chose a more general image format, bitmap, to facilitate later analyses. Of the bitmap formats, 24-bit color was chosen to maintain image quality. Image compression from 12-bit (0–4095) to 24-bit RGB color (3 × 8 bit) was done with software installed to the FV500. For fluorescent images of microtubules, the grayscale intensity of 0–4095 was converted linearly to green, 0–255, with red and blue zeroed. Similarly, the grayscale intensity in fluorescent images of actin filaments was converted to red, 0–255, with green and blue zeroed.

### 2.3. Cell Segmentation from Confocal Fluorescence Microscopy Images

Cell geometry was determined from a series of cross-sectional fluorescent cytoskeleton images using the region-growing method. The region of interest expands from a seed area based on predefined criteria [21] by assimilating adjoining voxels only with properties similar to those of the seed voxel, such as grayvalue or color. We modified the region-growing method to determine the 3D cell shape from fluorescence microscopy images of cytoskeletons using an in-house C-language program.

**Figure 1:**
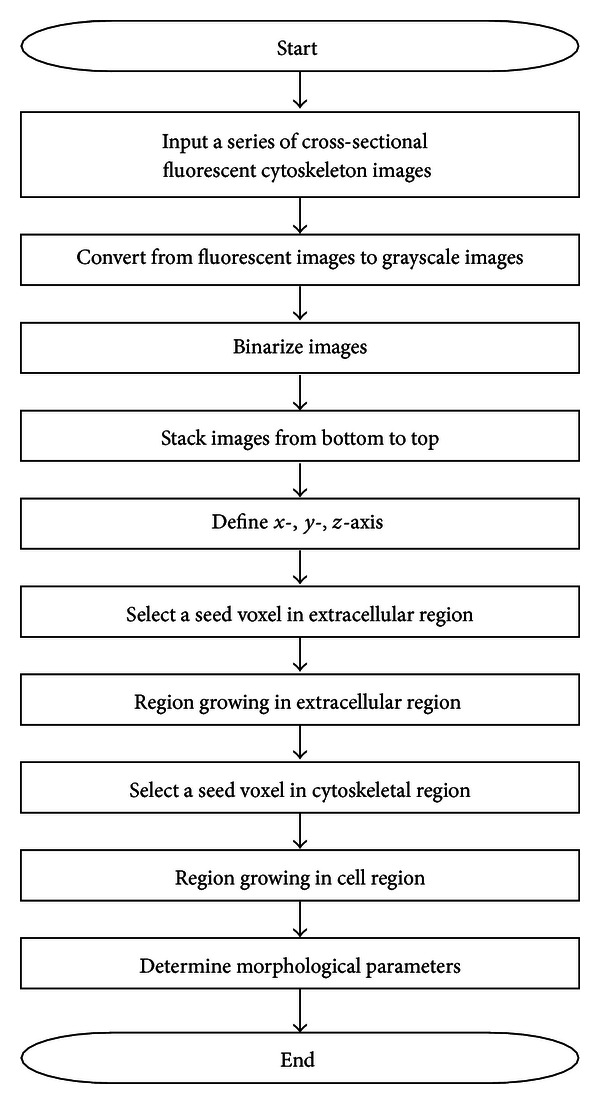
Flowchart of segmentation and morphometric analysis of cells from fluorescence microscopy images of cytoskeletons.

**Figure 2:**
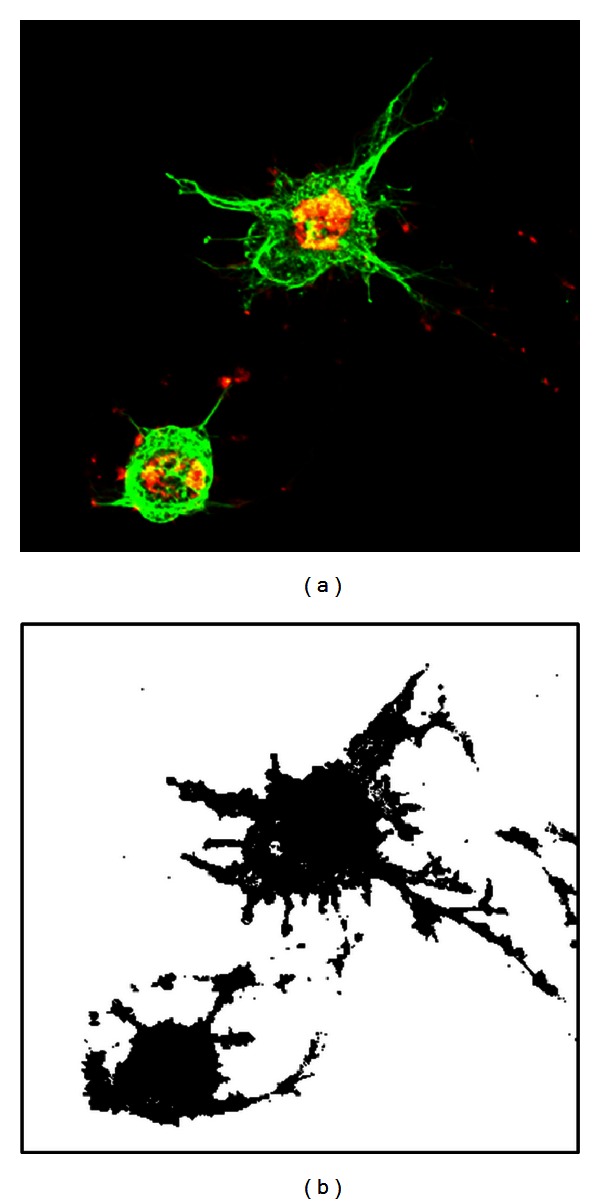
A height-wise superimposed fluorescence microscopy image of actin filaments (red) and microtubules (green) (left) and its binarized version, in which the cytoskeletons are black (right).

Figure 1 shows a flowchart of segmentation and morphometric analysis of cells from fluorescence microscopy images of cytoskeletons. Fluorescence microscopy images of cytoskeletons were doubly stained with the dyes fluorescein isothiocyanate and rhodamine, illuminated green, yellow, or red. Black pixels represented either the extracellular region or intracellular non-cytoskeletal regions, including the cytosol, nucleus, and organelles. No color-based criteria can differentiate the extracellular matrix and non-cytoskeletal regions. For subsequent image analyses, the originally 24-bit color formatted image was converted to a grayscale image using (1),

(1) $$\begin{array}{c} \text{GI}=0.3R_{\text{bv}}+0.59G_{\text{bv}}+0.11B_{\text{bv}}, \end{array}$$

where GI is the grayscale intensity and *R*_bv_, *G*_bv_, and *B*_bv_ are the RGB intensities ranging from 0 to 255. The images were binarized using a grayscale of 1 as the global threshold, so cytoskeletal regions appeared black and non-cytoskeletal regions white (Figure 2). To create a 3D cell image, the reformatted images were stacked from bottom to top to convert the pixels into voxels. Although confocal laser scanning microscopy has high-depth resolution, unfocused objects were imaged onto focused cross sections. Consequently, uncalibrated volume rendering of images resulted in height-wise stretching. We corrected this stretching using fluorescent spherical beads as a reference [12]. The *z*-axis was defined as the cell height with the bottom image set at *z* = 0. The *x*- and *y*-axes were set to form a right-hand Cartesian coordinate system. Voxels in cytoskeletal and non-cytoskeletal regions were then numbered 0 and 1, respectively.

**Figure 3:**
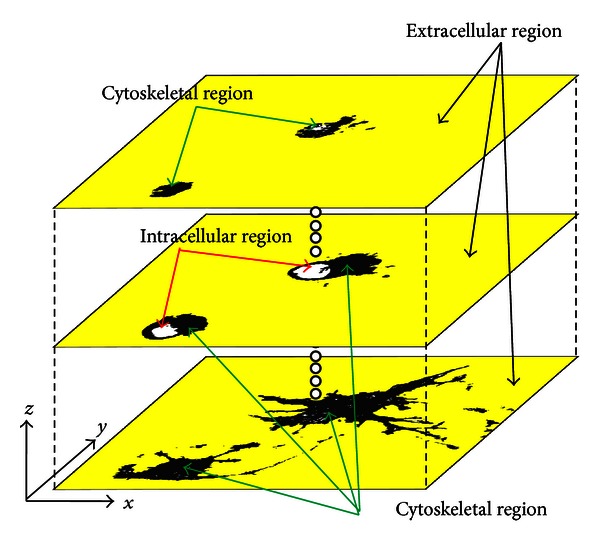
The extracellular region was determined by implementing region growing in all directions (*x*, *y*, *z*) from a seed voxel established outside the cell. The extracellular region is yellow, the cytoskeletal region is black, and the intracellular noncytoskeletal region is white.

Before cell segmentation, we identified the extracellular region by introducing a seed voxel in the non-cytoskeletal region. Note that non-cytoskeletal regions were present not only outside but also inside cells where nucleus and cytoplasmic components were present. Therefore, care was taken to not introduce the seed voxel inside cells. Regional growth was then applied from the seed voxel in all directions (*x*, *y*, *z*) to assimilate adjoining voxels flagged 1, non-cytoskeletal regions, from the seed voxel.  Figure 3 shows the result of this process whereby extracellular voxels of the non-cytoskeletal region are colored yellow with a flag of 2, while the rest is colored white with a flag of 3.

**Figure 4:**
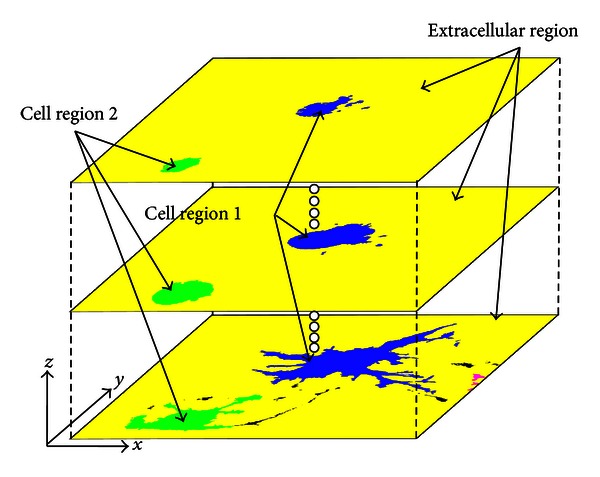
Results of cell segmentation using the region-growing method. For identification, the different cells are colored blue and green.

Finally, cells were identified using the region-growing method for voxels with a flag of 0, the cytoskeletal region, in all directions (*x*, *y*, *z*). The region was grown for voxels flagged either 0 or 3. Through this process, both cytoskeleton voxels and intracellular non-cytoskeleton voxels are assimilated to the seed voxel. To identify individual cells when multiple cells were in one image, the seed voxel of each cell was flagged with a unique number, and the voxels assimilated to the seed voxel were reflagged with the unique flag identifier. Cell identification was repeated until no voxels flagged with 0 or 3 remained in the images. Using this procedure, we segmented cells from a series of fluorescence microscopy images (Figure 4).

## 3. Results

**Figure 5:**
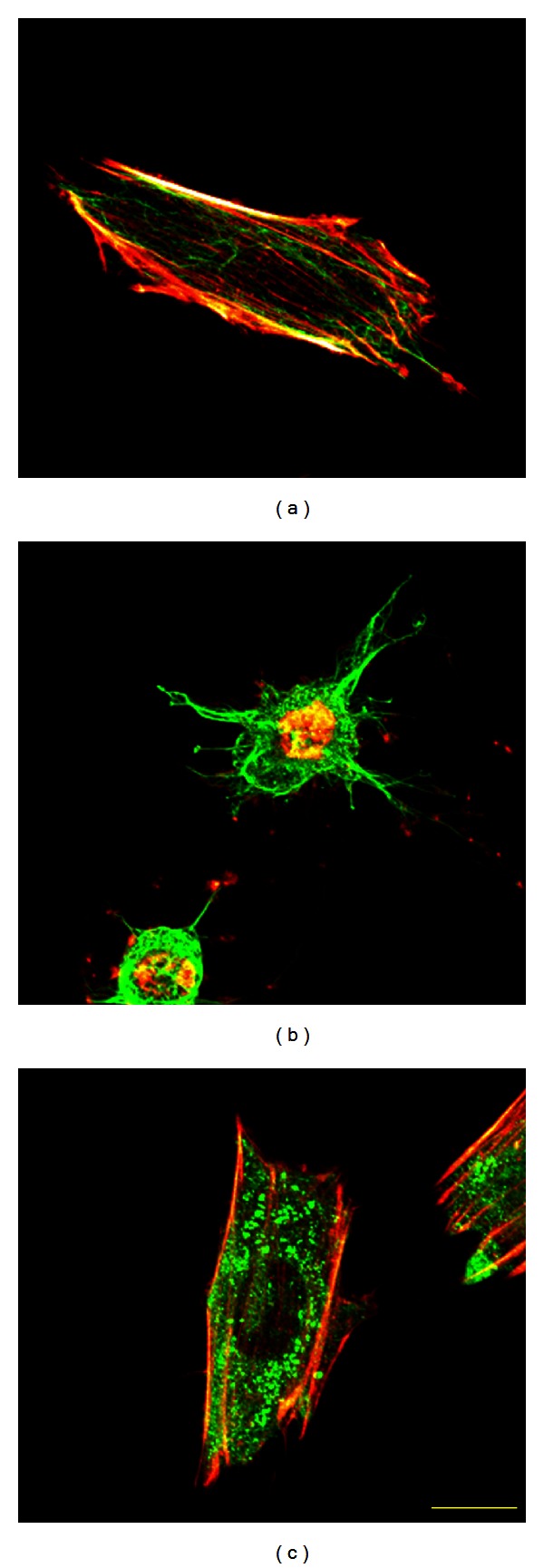
(a) Height-wise superimposed fluorescence microscopy images of actin filaments (red) and microtubules (green) of an intact cell (CON), (b) cells treated with cytochalasin D (CD), and (c) cells treated with colchicine (COL). Yellow bar = 20 *μ*m.

Figure 5 shows confocal laser scanning micrographs of actin filaments (red) and microtubules (green) in intact cells (CON) and cells treated with cytochalasin D (CD) or colchicine (COL). The *x-y* plane was superimposed on the *z*-axis in Figures 5(a)–5(c). Actin filaments and microtubules were observed throughout the cells, except in the nuclear region. Actin filaments appeared as thick actin bundles running parallel to the major axis of the CON cell, which was elongated elliptically in one direction (Figure 5(a)). Thick actin bundles were rarely seen in the CD cells, which had fragmented actin filaments gathered in the central region of the cell (Figure 5(b)). Microtubules were well preserved with thin processes protruding radially from the round cell body (Figure 5(b)). CD cells underwent drastic shape changes compared to CON cells (Figure 5(b)). While the microtubules of COL cells were almost completely fragmented, actin filaments remained whole (Figure 5(c)). Comparing CON and COL images revealed that microtubule disruption caused slight cell shrinkage.

**Figure 6:**
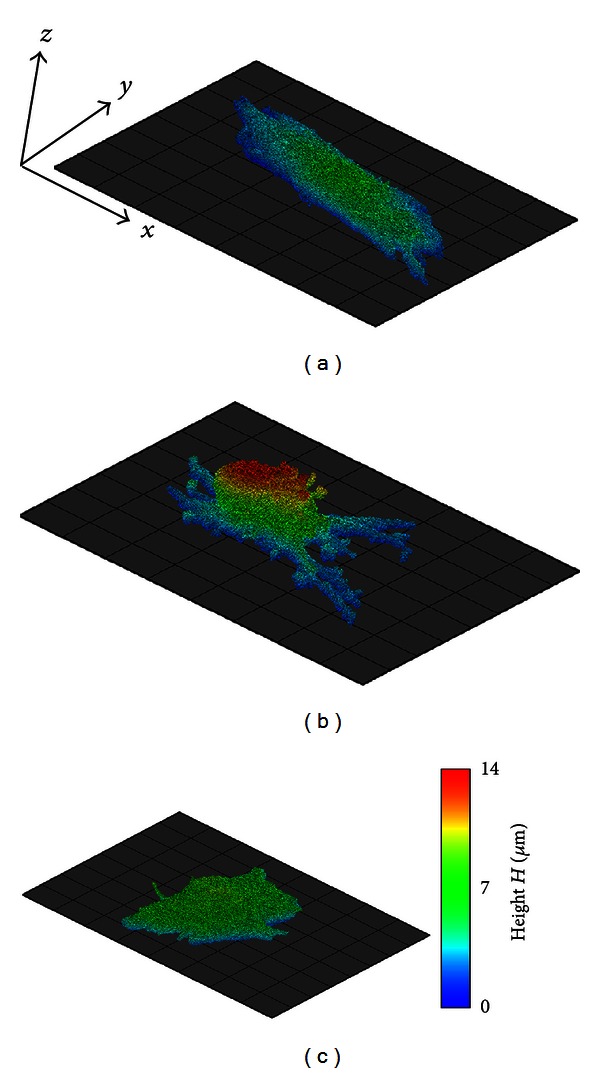
(a) Voxel images of cells segmented from fluorescence microscopy images of actin filaments and microtubules of an intact cell (CON), (b) a cell treated with cytochalasin D (CD), and (c) a cell treated with colchicine (COL). The grid base is 10 × 10 *μ*m. Color represents the *z*-coordinate of each voxel.

Figure 6 illustrates 3D reconstructions of the cells shown in Figure 5. We successfully segmented the complicated cell shapes in all groups. Comparing three-dimensionally reconstructed cells showed a drastic change in the morphological features of the CD cell (Figure 6(b)), which shrank lengthwise, while the height increased, resulting in a hemispherical body with some processes. The shape and height of the COL cell were similar to those of the CON cell (Figures 6(a) and 6(c)).

**Figure 7:**
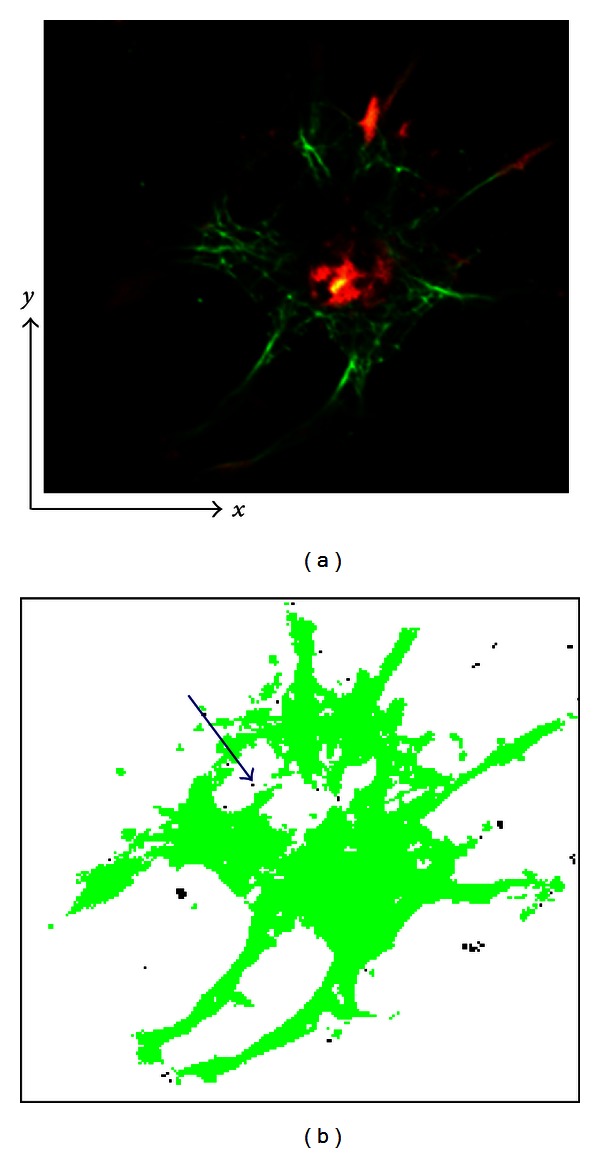
(a) A fluorescent image of cytoskeletons (red and green) on noncytoskeletal regions (black). (b) A cell image extracted using a conventional region-growing method in which a region is defined based on a difference in color or intensity.

Table 1 summarizes three morphological parameters of the cells extracted by a proposed method and a conventional region-growing method: volume, *V*, adhesion area, *S*_*a*_, and height, *H*. Statistical differences in each parameter of all groups were present between the proposed method and the conventional method. Especially, volume and adhesion area of cells extracted with the conventional method were significantly smaller than those extracted with the proposed method. This was because the conventional method failed to extract the voxels of intracellular non-cytoskeletal components as a part of the cell as shown in Figure 7. We used the Bonferroni correction to statistically compare these parameters among the three groups. A significant difference was not found between the groups when the proposed method was used, while CD and COL volume were significantly smaller than CON when the conventional method was applied. Cell adhesion area was ordered CON, COL, and CD, size-wise. Statistical differences in the adhesion area were present between both CON and COL (*P* < 0.05) and COL and CD (*P* < 0.05) obtained by both methods. CD height was significantly larger than CON and COL, whereas COL height did not differ statistically from CON obtained by both methods.

**Figure 8:**
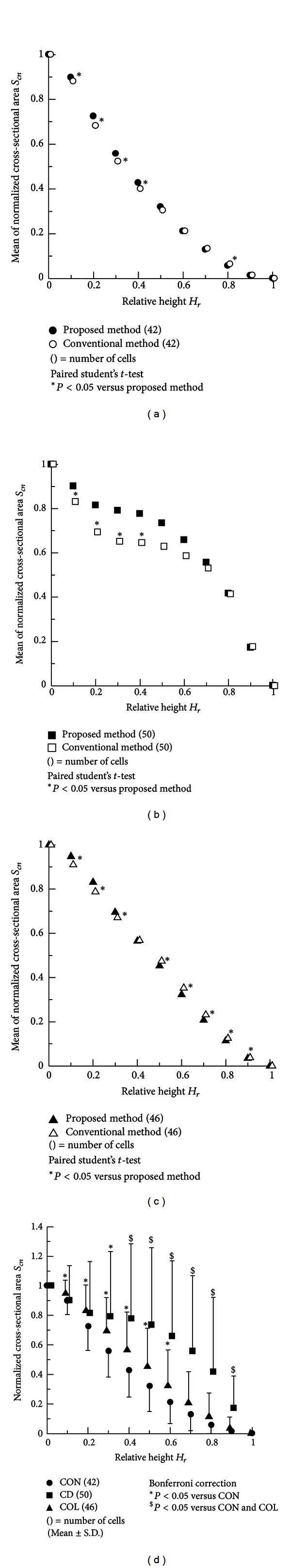
The cross-sectional area of cells normalized with adhesion areas (cross-sectional area at *z* = 0) in relation to their relative height *H*_*r*_. The mean of normalized cross-sectional area of (a) CON, (b) CD, and (c) COL extracted by a proposed method and by the conventional region-growing method. (d) A comparison of the normalized cross-sectional area of three groups extracted by a proposed method.

The mean cross-sectional area, *S*_*c*_, of each 10% increment of cell height was normalized using the adhesion area. The normalized cross-sectional area, *S*_cn_, was plotted against relative height (Figure 8). In all groups, the mean of the normalized cross-sectional area decreased from the bottom to the top. The mean of normalized cross-sectional area of CD groups obtained by the conventional method was significantly smaller than that obtained by the proposed method in the range of *H*_*r*_ = 0.1–0.6 although the mean of normalized cross-sectional area of CON and COL groups of the conventional method was nearly the same as those of the proposed method, respectively. As shown in Figure 8(d), the normalized cross-sectional areas of CD and COL extracted by the present method were significantly larger than those of CON in the range of *H*_*r*_ = 0.3–0.9 and 0.1–0.6, respectively (both *P* < 0.05, Bonferroni correction). The standard deviation of the CD normalized cross-sectional area was much greater than those of CON and COL.

## 4. Discussion

**Figure 9:**
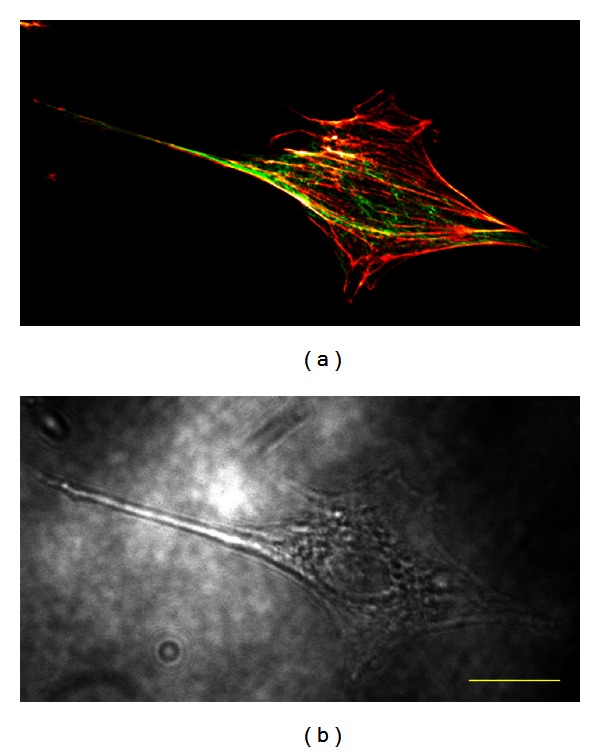
(a) A confocal laser scanning micrograph of actin filaments and microtubules and (b) an optical micrograph of the same cell. Yellow bar = 20 *μ*m.

Analyzing the effects of actin filament and microtubule disruption on cell morphology using simple parameters, including projection area and height, is difficult because of the 3D cell shape. For example, distinguishing among a circular cone, a cylindrical column, and an ellipsoidal body is impossible if they have the same projection area and height. We rebuilt 3D cell shapes using fluorescence images of actin filaments and microtubules. Although segmenting cells with fluorescence microscopy images of the cytoplasm and nucleus would presumably be easier and more accurate, we were interested in the relationship between cytoskeletal structures and cell shape, which necessitated cell shape segmentation from cytoskeleton images. Cytoskeleton outlines do not strictly coincide with the surface configuration of cells. As a result, we may have omitted cytoplasmic and other intracellular components, especially superficial ones, and underestimated cell volumes. Nevertheless, our pilot study confirmed that the cell outline delineated by cytoskeletons was almost the same as that observed using optical microscopy (Figure 9). Therefore, a cell segmented from fluorescence microscopy images of cytoskeletons represents cell geometry accurately enough to examine the mechanical role of cytoskeletons in maintaining cell shape.

**Figure 10:**
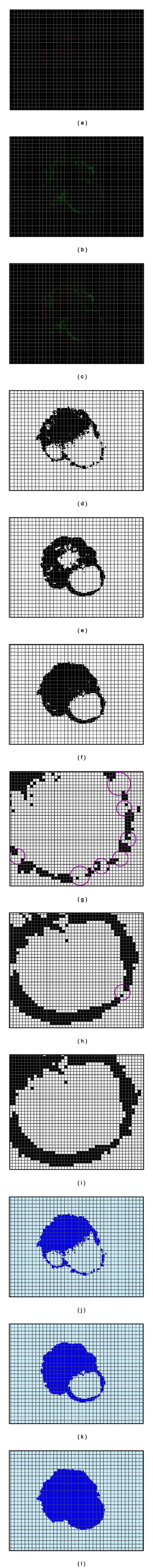
A fluorescent image of (a) actin filaments, (b) microtubules, and (c) both. Binarized images of the previous images (d)–(f) without and (g)–(i) with magnification. (j)–(l) Images of the extracted cell regions from (d)–(f) using the described method. The purple circles mark intracellular or nucleus regions not completely encompassed within the cytoskeleton.

Double-staining the cytoskeleton worked complementarily to help delineate cell shape. In the algorithm, regional growth is first implemented to define the extracellular region. However, the program fails when an intracellular region, especially the nuclear region where cytoskeletons are not present, is not perfectly isolated from extracellular regions by the cytoskeleton. The example in Figure 10 includes fluorescent images of actin filaments and microtubules separately and overlapped (a)–(c), binarized images of (a)–(c) without (d)–(f) and with magnification (g)–(i), and extracted cell regions of (d)–(f) using our method (j)–(l). When extracting cell regions from fluorescent images of either actin filaments or microtubules alone, the program falsely labeled the intracellular region as extracellular because it was not perfectly encompassed by the cytoskeleton (Figures 10(g) and 10(h)). To minimize the missing links, we overlapped the actin filament and microtubule fluorescent images (Figure 10(i)), with success (Figure 10(l)). Although this technique is not perfect, it drastically reduced the failure rate.

**Figure 11:**
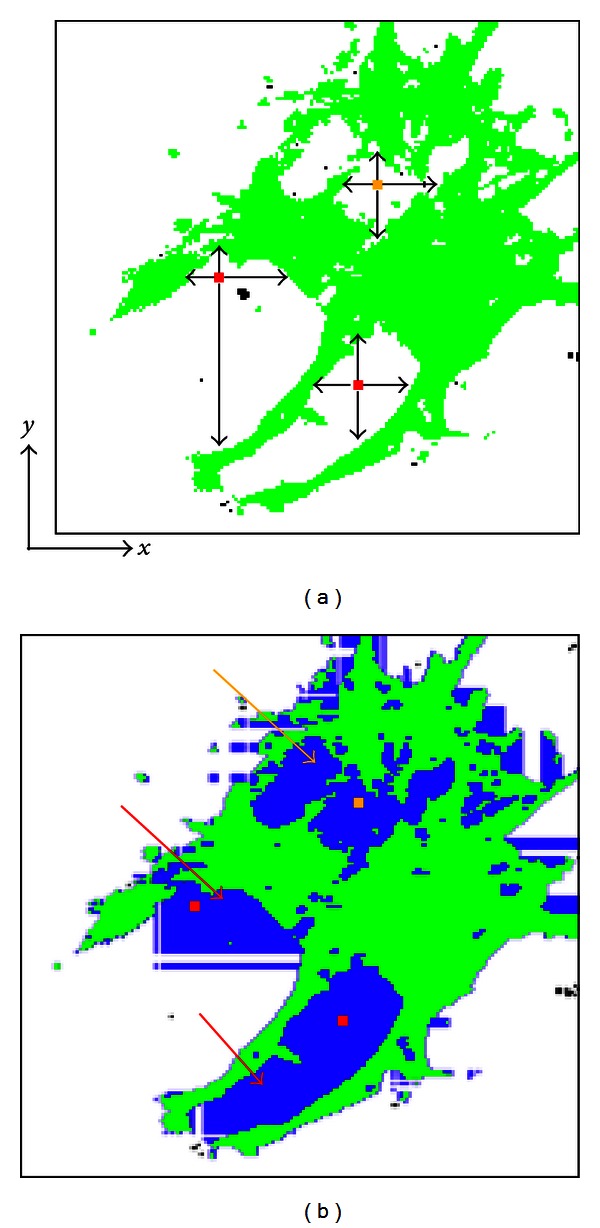
Differentiation of intracellular noncytoskeletal regions from the extracellular region. (a) Green regions represent the cytoskeletal pixels, while white ones represent the non-cytoskeletal pixels. The presence of cytoskeletal pixels surrounding (±*x*, ±*y*) a non-cytoskeletal pixel of interest was used to distinguish the intracellular non-cytoskeletal region from the extracellular region. If cytoskeletal pixels surrounded the pixel of interest on the* x-* and *y-*axes, it was recognized as an intracellular pixel. The yellow and red pixels are recognized as intracellular pixels, although red pixels should be identified as extracellular. (b) The resulting image of this process. Blue regions represent pixels recognized as intracellular as a result of this process. The red arrows show where this process incorrectly labeled some of the extracellular region.

The method was developed to reconstruct cell geometry using fluorescent cytoskeleton images. Conventionally, the region-growing method segments an object using image volumes. Voxels are grouped into larger regions by examining the properties of adjacent voxels radiating from a seed using predefined criteria [21], usually color intensity, including RGB and CT values [22]. Even using conventional methods, distinguishing objects of the same intensity or color is possible in terms of their domain, including the cytoplasm and nucleus [13, 20]. Nevertheless, since the voxels of intracellular non-cytoskeleton components and the extracellular matrix are black in fluorescence microscopy images (Figure 7(a)), differentiating them based solely on color intensity is impossible since only cytoskeletons are recognized as a part of the cell (Figure 7(b)). As a result, volume and adhesion area of cells extracted with the conventional method were significantly smaller than those extracted with the proposed method as shown in Table 1. The mean of normalized cross-sectional area of CD groups obtained by the conventional method was significantly smaller than that obtained by the proposed method as shown in Figure 8(b). These results were attributable to the deficiency of the conventional method; the conventional method failed to extract the voxels of intracellular non-cytoskeletal components as a part of the cell as shown in Figure 7. Therefore, a new algorithm was added, so that non-cytoskeleton pixels surrounded (±*x*, ±*y*) by cytoskeleton pixels were recognized as a part of the cell (Figure 11(a)). This algorithm was expected to distinguish the intracellular noncytoskeletal and extracellular regions. Although this process helped fill out the intracellular region, the resulting image included areas thought to be extracellular (Figure 11(b)). The method was revised to exclude pixels in the extracellular region from the previous process, which was accomplished by applying the region-growing method to the extracellular region. All nonextracellular, noncytoskeletal pixels must be intracellular and belong to one of the cells in the image. Applying the region-growing method to the extracellular region before the intracellular region is unique to this algorithm, as the region-growing method is normally used to segment only the region of interest.

**Figure 12:**
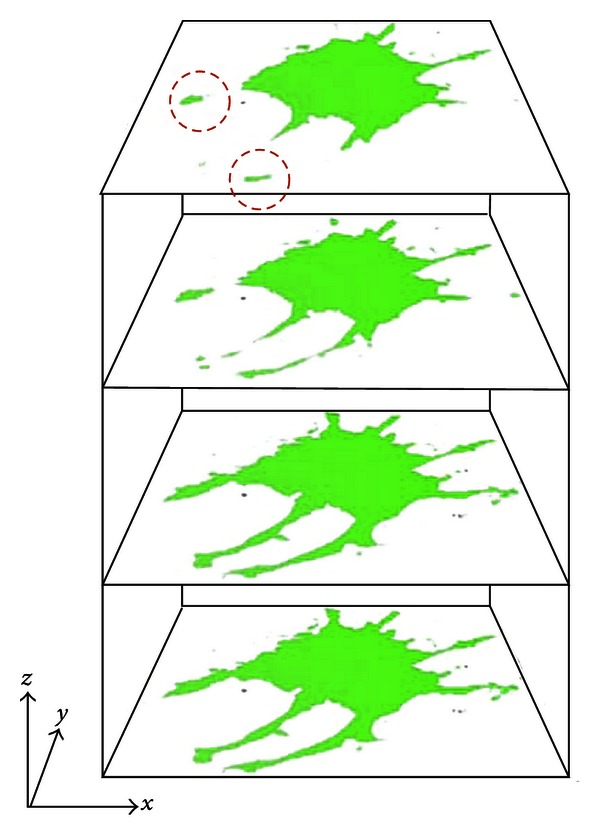
Cross-sectional images of a cell extracted using our method whereby the region was grown three-dimensionally. The red circles highlight the tips of parts of the cytoskeleton that appear to be separate from the main cell body in the top image. If the region were grown only in the *x*-*y* plane, these areas would be absent.

**Figure 13:**
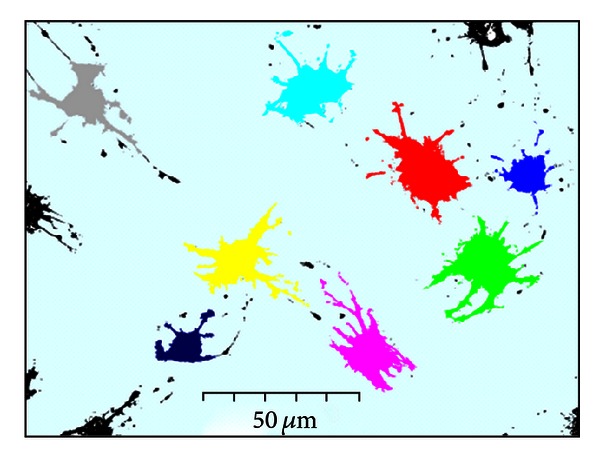
Cell segmentation obtained using our method. Eight cells in one image was identified individually using the program.

Regional growth was implemented three-dimensionally (Figure 12). The tips of three-dimensionally extending cytoskeletons appear isolated from the main body of the cell and would be absent if two-dimensional regional growth was performed on the top image. However, by expanding the region in the *z*, in addition to *x* and *y* directions, the three-dimensionally protruding processes were successfully extracted. This method facilitates the separate identification of each cell, even when multiple cells appear in one image (Figure 13). If cells are touching, they must be separated manually before running the program.

As observed in previous studies, disrupting actin filaments and microtubules induced 3D changes in cell shape [2, 7, 11]. In adherent cells, actin filaments generate contraction forces [23], and with focal adhesions, fasten the cell to the extracellular matrix. Therefore, the disruption of actin filaments leads to intracellular mechanical instability, causing partial detachment of the cell from the extracellular matrix at some focal adhesions [11, 24, 25]. In addition, a decrease in the actin filament contraction force induces a relative increase in cortical tensions. Therefore, the partial detachment and reduction in the contraction force of cells due to actin filament disruption results in an increase in cell height and decrease in cell adhesion area. In contrast to actin filaments, microtubules, together with the extracellular matrix, are thought to bear compressive forces that negate the contraction force at focal adhesions [11]. Therefore, microtubule disruption leads to a relative increase in contraction force, which overwhelms the compressive force exerted by the extracellular matrix.

The segmentation method we described has some limitations. It cannot be used on overlapping cytoskeletons of different cells because our algorithm examines only intensity-based flags of each voxel and cannot differentiate the cytoskeletons of two cells if they overlap. Hence, we excluded overlapping cells from the analysis. Another limitation lies in differentiating voxels of the extracellular matrix from those of intracellular components, which is problematic when cytoskeletons do not form a loop enclosing intracellular components. In such cases, intracellular voxels were merged with extracellular matrix voxels in the process of defining the extracellular region, which hollowed the cell body. Although these limitations must be addressed in future analyses, the morphometric data obtained suggest the potential of this method.

## 5. Summary and Conclusions

We developed a new method to reconstruct cell geometry from confocal fluorescence microscopy images of cytoskeletons. Extracting extracellular regions first to differentiate intracellular noncytoskeletal and extracellular regions, which both appear black in fluorescent images of cytoskeletons, is unique to this method. Cell geometry was successfully segmented from confocal fluorescence microscopy images of cytoskeletons, which outlined the cell. The analysis of the morphometric data revealed that actin filament and microtubule disruption lead to mechanical disequilibrium within the cell and at focal adhesions, causing the cell to reshape. The results indicate that this is a promising segmentation method for examining changes in cell morphological in relation to internal cytoskeletal structures. Using cell segmentation to reveal the cytoskeleton can be used to develop a more detailed cell structure model and to study the relationships among cell mechanics, functions, and structures.

## Figures and Tables

**Table 1:** The summary of the volume, adhesion area, and fibroblast height of the three cell groups.

	CON (*n* = 42)	CD (*n* = 50)	COL (*n* = 46)
Volume *V* (*μ*m^3^)			
Proposed method	2742 ± 1529	2165 ± 1151	2201 ± 949
Conventional method	2440 ± 1340*	1777 ± 917^∗#^	1811 ± 743^∗#^
Adhesion area *S*_*a*_ (*μ*m^2^)			
Proposed method	989 ± 480	357 ± 178^$^	722 ± 389^#^
Conventional method	902 ± 454*	320 ± 157^∗$^	609 ± 327^∗#^
Height *H* (*μ*m)			
Proposed method	7.4 ± 2.0	10.7 ± 1.6^$^	7.5 ± 2.4
Conventional method	7.5 ± 2.0*	10.7 ± 1.7^∗$^	7.5 ± 2.4*

*P < 0.05 versus proposed method (paired Student's *t*-test).

^#^ *P* < 0.05 versus CON, ^$^ *P* < 0.05 versus CON and COL (Bonferroni correction).
